# Lacrimal gland biopsies—results from a tertiary centre in the UK

**DOI:** 10.1038/s41433-022-02331-9

**Published:** 2022-12-21

**Authors:** Segun Awotesu, Ebube Obi, Mary Awad, Hardeep Mudhar, Joyce Burns, Ian De Silva, Raghavan Sampath

**Affiliations:** 1 Department of Ophthalmology, University Hospitals of Leicester, Infirmary Square, LE1 5WW Leicester, UK; 2 Department of Pathology, Royal Hallamshire Hospital, Glossop Road, S10 2JF Sheffield, UK

**Keywords:** Lacrimal apparatus diseases, Diseases, Eye diseases

## Abstract

**Purpose:**

To report the histopathological results of lacrimal gland biopsies over a 21-year period in a tertiary referral centre in the United Kingdom. To the best of our knowledge, this represents the largest series to be published in the United Kingdom.

**Methods:**

A retrospective observational review was carried out for patients who underwent lacrimal gland biopsies in a tertiary referral centre at the University Hospitals of Leicester, United Kingdom between the years of 2000 and 2021.

**Results:**

Lacrimal gland biopsies were performed on 248 patients during the specified 21-year period. They comprised 157 (63.3%) females and 91 (36.7%) males. The mean age at presentation was 50.8 years (range 15–94 years). The majority of patients were Caucasian (69.4%, *n* = 172) followed by Asians (25.0%, *n* = 62), African/Afro-Caribbean (4.8%, *n* = 12) and other ethnicities (0.8%, *n* = 2). The most common histopathological diagnosis was chronic inflammation dacryoadenitis (69.0%, *n* = 171) followed by lymphomas (15.3%, *n* = 38).

**Conclusion:**

Our study shows that chronic inflammation accounts for the majority of histopathological diagnosis followed by lymphoproliferative disorders.

## Introduction

It was previously postulated that almost half of lacrimal gland lesions were of epithelial origin and the remaining were non-epithelial, based on figures derived from Reese’s 1956 clinico-pathologic survey of 112 consecutive expanding lesions of the lacrimal gland [1]. Furthermore, it found that one-half of the epithelial tumours were malignant, and a large proportion of the non-epithelial tumours were malignant lymphomas.

A clinico-pathologic review by Shields et al. in 1989 showed that the majority of lacrimal gland lesions arose from the non-epithelial part of the lacrimal gland and only 28% were of epithelial origin and contradicted the aforementioned results [2]. Historically, there has been a shortage of publications on lacrimal gland biopsies undertaken in the United Kingdom. This study reviewed all lacrimal gland lesions that were biopsied during a 21-year period in a tertiary referral centre, at the University Hospitals of Leicester, UK. We aim to provide factual data about the origin and frequency of lacrimal gland lesions within a diverse UK population.

## Methods

A retrospective observational study of patients who underwent a diagnostic open sky approach lacrimal gland biopsy over a 21-year period from 2000 to 2021 was conducted. This included both incisional and excisional biopsies. Cases were identified from both our theatre and histopathology departmental electronic databases. Once identified, the medical records of the patients and their histological reports were obtained for analysis. Only intrinsic tumours of the lacrimal gland were included. Exclusion criteria included missing data in the case notes or incorrect patient details.

**Fig. 1:**
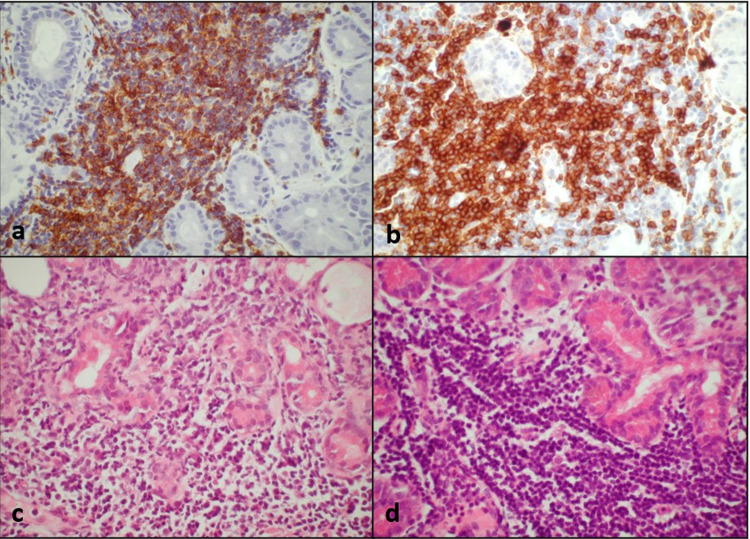
The histopathological appearance of lacrimal gland diffuse lymphoid infiltrate using various stains. **a** CD20 stain, **b** CD3 stain, **c** Hematoxylin & Eosin stain and **d** dense inflammatory infiltrate visualised with Hematoxylin & Eosin.

Data collected included socio-demographic variables, clinical symptoms and signs, and correlating imaging features. Tissue biopsy specimens were reviewed by a senior pathologist to confirm the histological diagnosis. In cases where there was a suspicion of lymphoproliferative disease or in cases of chronic inflammation, the specimens underwent further molecular genetic analysis including immune-phenotyping and gene rearrangement studies (Fig. 1).

## Results

Lacrimal gland biopsies were performed on 248 patients during the specified 21-year period. They comprised 157 (63.3%) females and 91 (36.7%) males. The mean age at presentation was 50.8 years (range 15–94 years). Most patients were Caucasian (69.4%, *n* = 172) followed by Asians (25.0%, *n* = 62), African/Afro-Caribbean (4.8%, *n* = 12) and other ethnicities (0.8% *n* = 2).

Table 1 highlights a summary of the results that were found in this study. The most common histopathological diagnosis was specific or non-specific chronic inflammation (dacryoadenitis), which accounted for 69.0% of cases (*n* = 171), followed by lymphomas (15.3%, *n* = 38). There was no link identified between ethnicity and biopsy findings from our data.

**Table 1:** A summary of the results of the study.

Sex		Ethnicity		Pathology		Inflammatory		Lymphoma	
Male	91	African	12	Inflammatory	171	Chronic	145	Extra-nodal marginal zone	12
Female	157	Asian	62	Lymphoma	38	Acute on chronic	3	Diffuse large B cell	9
		Caucasian	172	Reactive/lymphoid hyperplasia	2	Granulomatous (sarcoid)	7	Mantle cell	7
		Chinese	1	Epithelial cyst	7	Granulomatous (GPA)	1	Follicular	6
		Mixed	1	Epithelial tumour	14	Granulomatous (other)	6	Other	4
				Infection		Autoimmune (Sjogren’s)	4		
				Varicella	1	Vasculitis	2		
				TB	2	Infective	3		
				Other	13				

*GPA* granulomatosis with polyangiitis, *TB* tuberculosis.

Normal lacrimal gland was seen on histo-pathologic examination in two patients (0.8%) with prolapsed lacrimal glandular tissue. Seven biopsies (2.8%) showed lacrimal gland cysts with no dysplastic features.

Of the 171 patients reported to have dacryoadenitis on biopsy, 2 patients (1.2%) had evidence of small vessel vasculitis on histopathology and 4 patients (2.3%) had autoimmune Sjogren’s syndrome. One patient was diagnosed with granulomatosis with polyangiitis. Granulomatous inflammation secondary to sarcoidosis was seen in 7 patients (4.1%), and 1 patient (0.6%) was subsequently diagnosed with IgG4 disease. Two (1.2%) patients had an infective aetiology with a positive QuantiFERON^®^ gold test indicative of tuberculosis.

Lymphomas accounted for 15.3% (*n* = 38) of all lacrimal gland biopsies performed. The mean age in the lymphoma group was older than the overall cohort as expected with a mean age of 66 years. Sixteen (42.1%) of lymphoma cases were male and 22 (57.9%) of cases were female. Extra-nodal marginal zone B cell lymphoma was diagnosed in 12 biopsies (31.6%), diffuse large B cell lymphoma in 9 cases (23.7%), mantle cell lymphoma in 7 cases (18.4%), follicular lymphoma in 6 cases (15.8%), and 4 cases (10.5%) had a diagnosis of unspecified lymphoma. One patient with B cell lymphoma had typical histopathological features of chronic lymphocytic leukaemia on subsequent analysis.

Of the lymphoma group, seven were initially diagnosed as inflammatory, but on further subsequent cellular analysis were confirmed as lymphomas. Nearly half of patients with a positive lacrimal lymphoproliferative result had evidence of systemic lymphoma concurrently or subsequent to the diagnosis of lacrimal gland lymphoma. With targeted treatment, five patients showed complete remission of their orbital and systemic disease.

Pleomorphic adenomas were detected in 2.0% (*n* = 5) of all biopsies and adenocarcinomas in 3.6% (*n* = 9). Of the adenocarcinoma group, two had high-grade carcinoma ex pleomorphic adenoma, two had adenoid cystic carcinomas, one had a primary lacrimal gland ductal carcinoma, one had a suspected metastasis from oesophageal carcinoma and the rest were unspecified. All the pleomorphic adenomas were suspected on clinical and imaging findings prior to surgery, and had planned excision biopsies.

Pre-operative computed tomography (CT) finding predictive of lymphoma on biopsy included moulding of lesion to orbital structures. Presence of globe and extra-ocular muscle displacement, especially of the superior and lateral recti muscles, were also noted to be predictors of lymphoma. No specific radiological finding was predictive of a histopathological diagnosis of dacryoadenitis.

## Discussion

**Fig. 2:**
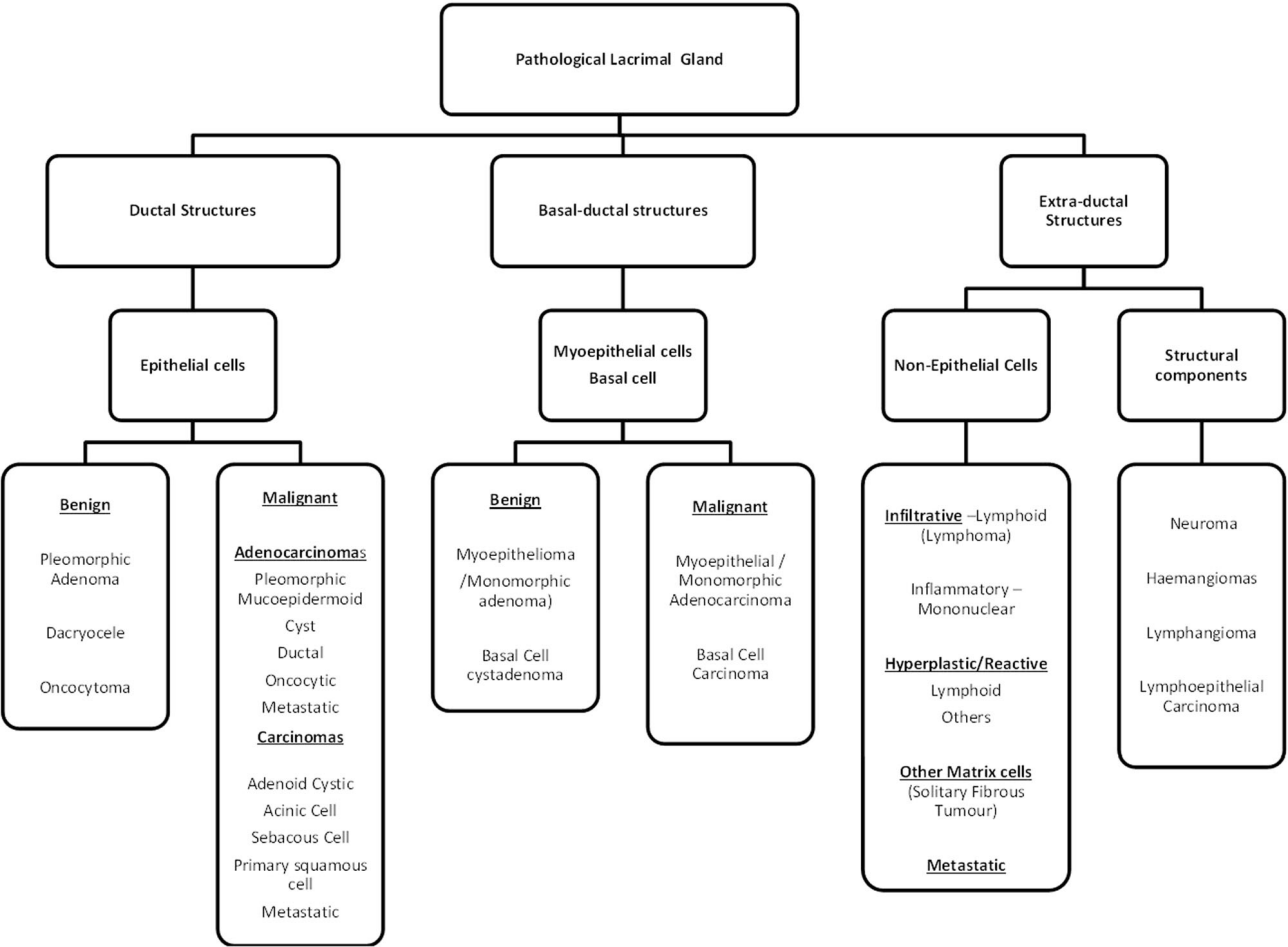
A summary of the origins of the lacrimal gland tissues and the types of pathology that can arise from each.

Lacrimal gland biopsies form an integral part in the diagnostic pathway of patients with lacrimal gland lesions. A normal lacrimal gland is composed of various cellular constituents, broadly classified into epithelial (glandular) and non-epithelial (non-glandular) components. Any of the cellular constituents within these components can give rise to various pathologies (Fig. 2).

Our study’s aim was to investigate the types of pathology that required an incisional or excisional lacrimal gland biopsy. Like other studies, it confirms that the majority of the biopsied specimens were of non-epithelial origin (see Table 2) [1–12].

**Table 2:** A summary list of clinico-pathological lacrimal gland studies published in the literature with the results of these study.

Author	Study size	Study site	Years studied	Epithelial pathology, number of cases (%)	Non-epithelial pathology, number of cases (%)	1st Most common pathology (number of cases)	2nd Most common pathology (cases)	3rd Most common pathology (cases)
Reese [1]	113	USA	Not specified	54 (48%)	59 (52%)	Inflammation (35)	Epithelial (B* = 27, M* = 27)	Lymphoma (24)
Font et al. [3]	120	Houston, USA	A 23-year period	41 (34%)	79 (66%)	Inflammation (75)	Pleomorphic adenoma (17)	Lymphoma (4)
Shields et al. [2]	142	Philadelphia, USA	1962–1987	32 (23%)	110 (77%)	Inflammation (90)	Lymphoid (B* = 13, M* = 6)	Pleomorphic adenoma (17)
LGTSG [4]	311	Various sites, Japan	1995–1997	131 (42%)	180 (58%)	Pleomorphic adenoma (86)	Lymphoma (55)	Inflammation (50)
Teo et al. [5]	69	Singapore	2000–2010	9 (13%)	60 (87%)	Inflammation (32)	Lymphoid (B* = 13, M* = 12)	Pleomorphic adenoma (7)
Elbakary et al. [6]	146	Egypt	A 10-year period	32 (22%)	114 (78%)	Inflammation (63)	Lymphoid (B* = 11, M* = 28)	Epithelial (M* = 13, B* = 9)
Ng et al. [7]	23	Hong Kong	2000–2006	7 (30%)	16 (70%)	Inflammation (9)	Lymphoid (B* = 4, M* = 3)	Epithelial tumours (M* = 1, B* = 5)
Wright et al. [8]	108	Moorfields, UK	1968–1979	40 (37%)	68 (63%)	Epithelial (M* = 20)		
Tang et al. [9]	74	USA/Australia	2005–2013	2 (3%)	74 (97%)	Inflammation (44)	Lymphoid (B* = 8, M* = 10)	Prolapsed normal gland (3)
Ahn et al. [10]	95	Korea	2010–2018	19 (20%)	76 (80%)	Inflammation (51)	Lymphoid (B* = 6, M* = 18)	Epithelial tumours (B* = 13, M* = 3)
Andrew et al. [11]	268	Australia	1997–2012	40 (15%)	222 (83%)	Inflammation (124)	Lymphoid (B* = 33, M* = 53)	Epithelial tumours (B* = 21, M* = 11)
Von Holstein et al. [12]	232	Denmark	1974–2007	88 (38%)	117 (50%)	Inflammation (62)	Epithelial (B* = 31, M* = 32)	Lymphoid (B* = 10, M* = 25)
Our study	248	Leicester UK	2000–2021	21 (8.5%)	227 (91.5%)	Inflammation (171)	Lymphoma (38)	Epithelial tumours (B* = 5, M* = 9)

M* malignant lesions, B* benign lesions.

*GPA* Granulomatosis with polyangiitis, *TB* Tuberculosis.

The predominant lacrimal gland lesion, of non-epithelial origin, was found to be inflammation. Seven patients that were originally diagnosed with chronic inflammation were subsequently reclassified to lymphoma following molecular genetic analysis and immuno-phenotyping.

It is important to note that IgG4-related disease may have been reported as chronic non-specific dacryoadenitis in some of the earlier biopsies. In 2004, Kamisawa discovered that patients with autoimmune pancreatitis also showed the involvement of organs other than the pancreas [13]. In 2007, Cheuk et al. suggested that the previous chronic sclerosing dacryoadenitis may be part of the spectrum of IgG4-related sclerosing disease [14]. Although known by different names, there was international consensus and recommendation to unify the term IgG4-related disease followed by the organ or area affected (IgG4-related dacryoadenitis) [15].

Lacrimal gland enlargement was the most common presenting symptom in our study. Tamboli et al. [16] examined lacrimal gland size in 300 Caucasian orbits. They found that size decreases with age, and that there were no gender differences. They demonstrated that the mean (10th and 90th percentiles) lacrimal gland axial length in the right orbits were 14.7 mm (10.9 and 18.3 mm) and 14.5 mm (10.3 and 18.3 mm) in left orbits. Coronal length averaged 17.7 mm in right eyes (13.9 and 21.8 mm) and 16.9 mm (12.8 and 20.8 mm) in left eyes. These ranges can help in differentiating diseased versus non-diseased lacrimal glands, especially in conjunction with physical examination. CT and magnetic resonance imaging (MRI) features of lacrimal inflammation include moderate to intense enhancement, along with lacrimal gland enlargement and scleritis in idiopathic orbital inflammation [17].

The wedge sign, describing a triangle of tissue between the lateral rectus muscle and lateral orbital wall and the superior rectus muscle and the orbital roof, has been noted to be a prognostic feature in lacrimal gland inflammation, lacrimal gland carcinoma, or lymphoma [18].

One of the limitations of a study such as this in a tertiary centre may be due to the nature of referrals from surrounding areas. Referrals to tertiary centres may increase the proportion of epithelial tumours seen. In this study, all regional lacrimal gland biopsies are typically referred to our tertiary centre and hence this would be more representative of the population. This may also be an explanation for why a greater proportion of epithelial tumours may have been found in other studies.

In this study, over half of biopsies were reported as chronic inflammation with no cause attributed to this. As discussed earlier, IgG4-related dacryoadenitis is a recognised cause of chronic inflammation. In future studies, it would be interesting to assess what proportion of these chronic inflammatory biopsies could be attributed to IgG4-related disease with or without systemic involvement.

Equally, looking at the presence of other systemic autoimmune diseases in these cases would be useful to assess if this may be a predictor for chronic inflammatory dacryoadenitis.

## Conclusion

Lacrimal gland biopsies may present a plethora of pathologies some of which may have grave implications; however, chronic inflammation remains the major pathological finding.

### Summary

#### What was known before

- Previous publications on lacrimal gland biopsies were equivocal in their findings of the proportion of epithelial versus non-epithelial origins.

#### What this study adds

- Proportion of lesions of epithelial origin was found to be less than 10%.
- Chronic inflammation accounted for the majority of biopsy results.
- Extra-nodal marginal zone lymphoma was found to be the most frequent subtype of lymphoma identified.

## Data Availability

The datasets generated during and/or analysed during the current study are available from the corresponding author upon reasonable request.
